# Association between HLA-DRB1 polymorphisms and pemphigus vulgaris: a meta-analysis

**DOI:** 10.1111/j.1365-2133.2012.11040.x

**Published:** 2012-10

**Authors:** L Yan, J-M Wang, K Zeng

**Affiliations:** Department of Dermatology, Nanfang Hospital, Southern Medical UniversityBaiyun District, Guangzhou, Guangdong Province, 510515, China

## Abstract

**Summary:**

*Background* Several studies have reported that HLA-DRB1 may be correlated with pemphigus vulgaris (PV), but most have been based on small samples and the results remain inconsistent and unclear.

*Objectives* To investigate the correlation between DRB1 and PV by a meta-analysis of case–control/nonfamily studies.

*Methods* PubMed, Wiley Online Library, ScienceDirect, Google Scholar, Cochrane Library, Chinese National Knowledge Infrastructure and Wanfang databases were searched for studies including: (i) ‘pemphigus’; and (ii) ‘human leukocyte antigen’, ‘HLA’, ‘major histocompatibility complex’, ‘MHC’ or ‘DRB1’. Eighteen selected studies were used in meta-analyses to evaluate DRB1 alleles and phenotypes by calculating the respective odds ratios (ORs) and 95% confidence intervals (CIs). Stratified meta-analyses and meta-regression analysis were also conducted.

*Results* The frequencies of three genotypes (allele and phenotype, respectively) were significantly increased in PV: DRB1*04 [*P*-value for comparability (*P*c) < 0·00001, OR 3·61, 95% CI 2·28–5·71; *P*c = 0·0002, OR 4·14, 95% CI 1·98–8·65], DRB1*08 (*P*c = 0·03, OR 2·25, 95% CI 1·07–4·70; *P*c = 0·0003, OR 2·46, 95% CI 1·51–4·01) and DRB1*14 (*P*c < 0·00001, OR 6·47, 95% CI 4·52–9·26; *P*c < 0·00001, OR 9·68, 95% CI 4·47–20·98). Three others (allele and phenotype, respectively) were significantly decreased in PV: DRB1*03 (*P*c < 0·00001, OR 0·28, 95% CI 0·19–0·41; *P*c = 0·0001, OR 0·25, 95% CI 0·12–0·51), DRB1*07 (*P*c = 0·004, OR 0·45, 95% CI 0·26–0·78; *P*c = 0·0002, OR 0·27, 95% CI 0·14–0·54) and DRB1*15 (*P*c = 0·001, OR 0·35, 95% CI 0·18–0·66; *P*c = 0·002, OR 0·32, 95% CI 0·16–0·65). Ethnicity partially explained the heterogeneity of DRB1*07, DRB1*08 and DRB1*14 phenotypes.

*Conclusions* Our findings suggest that DRB1*04, DRB1*08 and DRB1*14 are statistically significant susceptibility factors for PV. Conversely, DRB1*03, DRB1*07 and DRB1*15 may be negatively associated with PV. Specific HLA-DRB1 types may influence the susceptibility or resistance to PV, which needs further investigations.

Pemphigus is a rare and sometimes fatal autoimmune bullous disorder that manifests as flaccid, painful, nonhealing blisters or ulcerations on the skin and mucosal surfaces.^1^ Histologically, pemphigus is characterized by the presence of epidermal acantholysis, as a result of circulating and tissue-fixed antibodies against keratinocyte surface antigens, separation of desmosomes and dissolution of the intercellular material. If left untreated, the resulting widespread bullae and erosions can increase the probability of infection, aggravate metabolic disturbance, and trigger an inappropriate and detrimental immune response. The disease ordinarily requires long-term therapy, most often with systemic corticosteroids;^2^ however, these treatments are associated with serious side-effects.^3^ Thus, there is an urgent need to gain a detailed understanding of the aetiology and pathogenesis of pemphigus.

Pemphigus onset usually occurs between the 4th and 6th decades of life and has been reported in various populations. Worldwide annual incidence has been estimated at 0·76–6·7 cases per million.^3^ Three basic forms of pemphigus are currently recognized, based on distinctive clinical signs and pathology: pemphigus vulgaris (PV), pemphigus foliaceus and paraneoplastic pemphigus. PV is the most commonly diagnosed form, and accounts for approximately 80% of all pemphigus cases. In PV, mucous membranes are affected and the circulating autoantibodies mainly target the desmosomal cadherin family member, desmoglein (Dsg) 3. It has been demonstrated that Dsg3-reactive T and B cells both indirectly regulate formation of pathogenic autoantibodies.^4^ The presence of IgG1 and IgG4 against Dsg3 was directly related to the ratio of Dsg3-reactive Th1/Th2 cells,^5^ and autoreactive T cells, that recognized epitopes of the extracellular domain of Dsg3, were restricted by PV-associated human leucocyte antigen (HLA)-DRB1 alleles.^6^^,^^7^ Furthermore, autoimmune responses to Dsg3 may be strictly regulated by the hydrophobic amino acid residues Phe^26^, Leu^67^ and Val^86^ and hydrophilic amino acid residues at positions 70 and 71 on the HLA-DR β chain.^8^

The demonstrated association of PV with HLA class II has been supported by serology, genotyping and sequencing in cases from different ethnicities. For example, alleles of HLA-DR4 (DRB1*0402 and *0406 in Ashkenazi Jews^9^ and Asians,^10^^,^^11^ respectively) and DR14 (DRB1*1401 and *1405 in Europeans^12^^,^^13^ and Asians,^10^^,^^14^ respectively) confer strong susceptibility to PV in different ethnic populations. Nevertheless, the association between some HLA-DRB1 genes and PV remains controversial and obscure in some population-based studies. One such study demonstrated that the autoantibody response in Jewish patients with PV was linked to HLA-DR4, but in non-Jewish patients of European ancestry it was linked to DR6.^12^ DRB1*07^15^ was associated with increased susceptibility to PV in Italians (southern Europe) while a similar observation had already been reported for DRB1*13 in Spanish patients.^16^ Interestingly, Korean patients with PV have remarkably high frequency of DRB1*01^17^ rather than DRB1*04 or *14. In contrast, individuals of Moroccan descent appear to benefit from a unique protective effect of DRB1*15.^18^

It is possible that these seemingly ethnic-specific roles for HLA-DRB1 are merely the product of statistical bias introduced by the relatively small sample sizes in each individual report. As such, a meta-analysis research method may be able to reveal more precisely the true role of HLA-DRB1 genes in PV. Here, we describe the first meta-analysis study of HLA-DRB1 as related to PV susceptibility or resistance. The findings from our study provide further insights into the underlying mechanisms of PV pathogenesis and may help future development of effective therapies to prevent and treat PV.

## Materials and methods

### Identification of relevant studies and data extraction

This study was designed according to the Meta-analysis Of Observational Studies in Epidemiology guidelines.^19^ We searched PubMed, Wiley Online Library, ScienceDirect, Google Scholar, Cochrane Library, Chinese National Knowledge Infrastructure and Wanfang database for studies associated with the following Medical Subject Heading terms: (i) ‘pemphigus’; and (ii) ‘human leukocyte antigen’, ‘HLA’, ‘major histocompatibility complex’, ‘MHC’ or ‘DRB1’. No restrictions were placed on language, patient age, sex or study design. All publications were searched up to 30 September 2011. In addition, the reference lists of all electronically identified studies and reviews were manually searched. All referenced dissertations and digests of conferences were retrieved. If additional studies or obscure data were suggested in any of the retrieved literature, the cited experts were contacted to obtain any additional sources of relevant data.

Two investigators (L.Y. and J-M.W.) independently reviewed all abstracts for study relevance, retrieved the selected full texts, extracted data using a standardized form, and assessed study quality. Disagreements were resolved by discussion and by consulting a third investigator (K.Z.). Studies were selected according to the following inclusion criteria: (i) an independent case–control design; (ii) primary study (not re-analysis or review) in English or Chinese; (iii) sufficient information to calculate the odds ratio (OR); (iv) unrelated cases and controls; (v) molecular-type detection methods (including high or low resolution level); and (vi) definite diagnosis of PV, based on histopathology or immunopatho-logy. In addition, studies were excluded based on the following features: (i) incomplete raw data; (ii) repetitive reporting of a dataset (for which, the most recent report was selected for inclusion); (iii) insufficient materials and methods; (iv) exclusive focus on haplotype or single nucleotide polymorphism; or (v) exclusive focus on HLA-DR4 or DR14.

The following information was collected from each of the selected studies: authors, publication year, ethnicity of study population, detection assay type, PV diagnostic criteria, and numbers of cases and controls for each genetic variation. The genetic data were categorized as allelic data (allele counts of a locus in the certain diploid species) or phenotype data (number of individuals positive for a given allele, regardless of heterozygosity or homozygosity).

### Quality assessment for included studies

In consideration of the various and complex nature of observational studies, we evaluated the internal validity of each selected article from different aspects. After referring to the treatise of Chalmers *et al.*^20^ and making minor modifications, we determined a unified form for assessing study quality that comprised four basic elements. First, the quality evaluation of ‘subjects’ was based on whether or not (i) selection criteria were used for the PV cases, including a specific definition of the diagnosis; and (ii) the control group was composed of individuals who were healthy and unrelated to the PV cases. Second, ‘comparability’ between PV cases and controls was judged according to statistical similarity in age, sex, ethnicity and area of residence. Third, the assessment of study ‘outcome’ was based on whether or not (i) the author mentioned use of blinding methods during the steps of data collection or interpretation of results; (ii) the results were clearly presented (with ORs and *P*-values); (iii) the results were comprehensive and objective (with negative results included); and (iv) a statement about regular heredity or Hardy–Weinberg equilibrium testing was included. Each paper was scored for these criteria on a scale of 0–10, with ≥ 5 indicating high quality and < 5 indicating low quality.

### Statistical analysis

The effect of DRB1 in PV was measured by using the Mantel–Haenszel method to calculate the ORs and 95% confidence intervals (CIs). Initially, numerical data were extracted directly and frequency data were multiplied by the total sample number and converted to a numerical statistical value.^21^ Then, Review Manager software (RevMan 5.0; available from the Cochrane Collaboration; http://www.cochrane.org) was used to conduct the meta-analysis. The heterogeneity among different studies was estimated by the χ^2^ test, the Q-statistic test and the *I*^2^ test. If the *P*-value for heterogeneity (*P*h) was > 0·10 or *I*^2^ < 50%, then heterogeneity was statistically insignificant^22^ and the fixed-effects model was applied; otherwise, the random-effects model was used. If the *P*-value for comparability (*P*c) was < 0·05 (two-tailed test) and the 95% CI of OR was exclusive of 1·0, then the result was statistically significant. In order to avoid type II errors, Bonferroni adjustment of the *P*-values was not performed for multiple comparisons.^23^^,^^24^ To test the robustness of the results, sensitivity analysis was performed by removing a single study at a time.

In addition, the two categorical results of a selected gene were comparatively analysed. If both results were consistent in the direction of 95% CI and had *P*c-value ≤ 0·05 but high heterogeneity, then subgroup (by RevMan 5·0) and meta-regression^25^ analyses were conducted to identify any potential sources of heterogeneity. The indices of stratified meta-analyses were ethnicity and quality classification, while covariates of meta-regression analyses were publication year, ethnicity and quality classification. The meta-regression analysis was performed with the STATA/SE 11.2 software (http://www.stata.com).

### Evaluation of publication bias

Funnel plot analysis was carried out by evaluating the symmetry of a scatter diagram. As more than five studies and experiential interpretation are required, we used Begg’s rank correlation test and Egger’s linear regression test^24^^,^^26^ to measure the degree of symmetry on quantified indices and to confirm the plot accuracy. The Begg’s and Egger’s tests were carried out by STATA/SE 11.2 software.

## Results

### Characteristics of included studies

**Fig 1:**
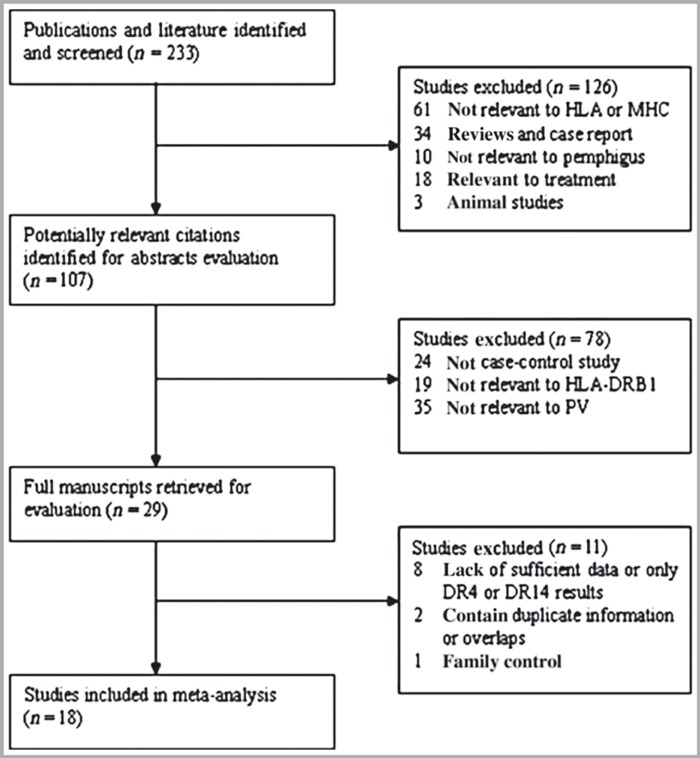
Flow chart of article selection process. HLA, human leucocyte antigen; MHC, major histocompatibility complex; PV, pemphigus vulgaris.

In total, 233 nonrepetitive articles were identified by the initial database search. After restrictive selection according to the inclusion and exclusion criteria (Fig. 1), 18 studies were selected for inclusion in the meta-analysis. The study characteristics are presented in Table 1. One letter to the editor by Saha *et al.*^27^ was included in the meta-analysis, despite lacking a description of diagnosis, as the patient information was provided by the PV Network (http://www.pemphigus.org.uk/). A variety of ethnicities was represented by the 18 studies: Asians,^8^^,^^10^^,^^14^^,^^17^^,^^28^ *n*=5; caucasians,^12^^,^^15^^,^^16^^,^^27^^,^^29^^–^^32^ *n* =8; other nationalities,^9^^,^^12^^,^^27^^,^^33^^–^^35^ *n* =6; mixture of caucasians and noncaucasians,^13^*n* =1. In addition, the 18 studies collectively provided data for 27 comparisons between different DRB1 types and PV.

**Table 1:** Characteristics of studies included in the meta-analysis

Study: first author, year of publication	Ethnicity	No. of PV patients (M/F), age in years	No. of controls (M/F), age in years	Diagnostic criteria of PV	No. of DRB1 alleles studied	HLA genotyping method	Data form
Ahmed, 1990^9^	Ashkenazi Jewish	26 (–/–), ND	59 (–/–), ND	Presence and biopsy	7	Lymphocyte microcytotoxicity assay and cDNA probes	AF and AC
Ahmed, 1991^30^	Non-Jewish caucasian	25 (–/–), ND	1000 (–/–), ND	Presence, DIF and IIF	3	Lymphocyte microcytotoxicity assay, RFLP and cDNA probes	AF and AC
Niizeki, 1994^14^	Japanese	32 (–/–), ND	45 (–/–), ND	Immunoblot assay	8	PCR-RFLP	PF and PC
Lombardi, 1996^29^	Italian non-Jewish	33 (–/–), ND	102 (–/–), ND	Clinical, histological and immunopathological	10	PCR-SSOP	PF and PC
Carcassi, 1996^32^	Sardinian and Italian	S: 16 (–/–), ND; I: 16 (–/–), ND	S: 296 (–/–), ND; I: 510 (–/–), ND	Clinical, histological and immunopathological	S: 3; I: 4	Mono-oligospecific alloantisera, RFLP and cDNA probes	AC, AF and PC, PF
Delgado, 1997^12^	Pakistani and non-Jewish European	P: 19 (–/–), ND; E: 19 (–/–), ND	P: 13 (–/–), ND; E: 248 (–/–), ND	Presence and IIF	P: 9; E: 9	PCR-SSOP	AC and AF
Lee, 1998^17^	Korean	15 (–/–), ND	100 (–/–), ND	Clinical, histological and immunopathological; some immunoblot assays	13	PCR-SSP	PF and PC
Gonzalez-Escribano, 1998^16^	Spanish caucasian	26 (10/16), ND	200 (–/–), ND	DIF	12	PCR-SSOP	PF and PC
Miyagawa, 1999^8^	Japanese	36 (–/–), ND	525 (–/–), ND	Histopathological, DIF and IIF	4	PCR-RFLP	PF and PC
Lombardi, 1999^15^	Italian caucasian	61 (–/–), ND	128 (–/–), ND	Clinical, histopathological and DIF	12	PCR-SSP	AC and AF
Loiseau, 2000^13^	French (caucasian, Asian and North African)	37 (16/21), 50·5 ± 14	106 (–/–), ND	Clinical, histological, DIF and IIF	3	PCR-SSOP	AC and AF
Zhou, 2003^10^	Chinese	61 (35/26), 51·48 ± 15·36	57 (35/22), 53·17 ± 15·93	Clinical, histopathological and DIF	14	PCR-SSP	AC and AF
Veldman, 2004^31^	German	14 (–/–), ND	11 (–/–), ND	Histopathological, DIF and IIF	9	PCR/ELPHA	AC, AF and PC, PF
Geng, 2005^28^	Chinese	27 (11/16), (19–69)	262 (–/–), ND	Clinical, histopathological and immunopathological	13	PCR-SSP	AC and AF
Sáenz-Cantele, 2007^33^	Venezuelan	49 (–/–), ND	101 (34/67), 36 ± 12	Clinical, histological and DIF	12	PCR and protocols, primer pairs and probes	AF and AC
Shams, 2009^34^	Iranian non-Jewish	52 (–/–), ND	180 (–/–), ND	Clinical, histological and DIF	13	PCR-SSP	AC and AF
Saha, 2010^27^	Caucasian European and Indo-Asian	E: 96 (–/–), ND; IA: 57 (–/–), ND	E: 100 (–/–), ND; IA: 59 (–/–), ND	ND	5	PCR-SSP	AC and AF
Tunca, 2010^35^	Turkish	25 (14/11), (20–69)	113 (65/48), ND	Clinical, histopathological and immunofluorescence microscopy	15	PCR-SSP	PF and PC

PV, pemphigus vulgaris; HLA, human leucocyte antigen; ND, not described; DIF, direct immunofluorescence; IIF, indirect immunofluorescence; RFLP, restriction fragment length polymorphism analysis; PCR, polymerase chain reaction; SSOP, sequence-specific oligonucleotide probes; SSP, sequence-specific primers; (–/–), no data mentioned in the included studies; ELPHA, enzyme-linked probe-hybridization assay; S, Sardinian; I, Italian; P, Pakistani; E, European; IA, Indo-Asian; AC, allele counts; AF, allele frequencies; PC, phenotype counts; PF, phenotype frequencies.

### Quality assessment of included studies

Table 2 shows the results of quality assessment for each of the 18 included studies. In three of the studies, homozygous cell lines from different ethnic groups were used as controls in lieu of individuals.^9^^,^^29^^,^^30^ None of the studies included the blinding methods (either during data collection or during interpretation of results) in their analyses. Moreover, no study considered the prevalence of each variant in the general population. Finally, 11 studies^8^^,^^10^^,^^12^^,^^15^^–^^17^^,^^28^^,^^32^^–^^35^ were defined as high quality, while the rest^9^^,^^13^^,^^14^^,^^27^^–^^31^ were defined as low quality.

**Table 2:** Quality assessment and scoring of included case–control studies

Study: first author, year of publication	Subjects		Comparability				Outcome			(6)	Score
	(1)	(2)	Age	Sex	Ethnicity	Region	(3)	(4)	(5)		
Ahmed, 1990^9^	*							*	*		3
Ahmed, 1991^30^	*							*			2
Niizeki, 1994^14^	*	*						*	*		4
Lombardi, 1996^29^	*							*	*		3
Carcassi, 1996^32^	*	*			*	*		*			5
Delgado, 1997^12^	*	*			*	*		*	*		6
Lee, 1998^17^	*	*			*	*		*	*		6
Gonzalez-Escribano, 1998^16^	*	*			*	*		*	*		6
Miyagawa, 1999^8^	*	*			*	*		*			5
Lombardi, 1999^15^	*	*			*	*		*	*		6
Loiseau, 2000^13^	*	*				*		*			4
Zhou, 2003^10^	*	*	*	*	*	*		*	*		8
Veldman, 2004^31^	*	*									2
Geng, 2005^28^	*	*	*	*	*	*		*	*		8
Sáenz-Cantele, 2007^33^	*	*				*		*	*		5
Shams, 2009^34^	*	*				*		*	*		5
Saha, 2010^27^		*			*	*		*			4
Tunca, 2010^35^	*	*		*		*		*	*		6

Asterisks indicate criteria that were satisfied. (1) Patient selection criteria, including specific definition of the diagnosis; (2) representative control group (unrelated and healthy individuals); (3) blinding during data collection and interpretation; (4) study results clearly presented (odds ratio, *P*-value); (5) reporting of negative results (lacking statistical significance); (6) the prevalence of each variant in the general population (Hardy–Weinberg law of genetic equilibrium).

### Meta-analyses of DRB1 polymorphism correlation to pemphigus vulgaris

The susceptibility or resistance correlations of HLA-DRB1 and PV are summarized in Tables 3 (alleles) and 4 (phenotypes). Pooled ORs and 95% CIs indicated that three HLA variants (DRB1*04, DRB1*08 and DRB1*14) were associated with a significant increase in risk of PV. In contrast, the frequencies of DRB1*03, DRB1*07 and DRB1*15 were significantly decreased in PV (*P* ≤0·05), suggesting a protective role.

**Table 3:** Summary of associations between HLA-DRB1 alleles and pemphigus vulgaris

HLA-DRB1 type	Studies	Allele counts		Heterogeneity			Model	Association			Sensitivity analysis	Publication bias		
		Patients (*n* events/*n* total)	Controls (*n* events/*n* total)	χ^2^	*P*h	*I*^2^%		*P*c	OR	95% CI		Funnel plot	Begg’s test (*P*r)	Egger’s test (*P*)
^*^01	^9^^,^^10^^,^^12^^,^^13^^,^^15^^,^^28^^,^^31^^,^^33^^,^^34^	27/730	166/2330	6·91	0·55	0	Fixed	0·002	0·50	0·33–0·77	None	S	0·902	0·795
^*^03	^9^^,^^10^^,^^12^^,^^15^^,^^27^^,^^28^^,^^31^^–^^34^	35/988	299/2690	8·65	0·47	0	Fixed	< 0·00001	0·28	0·19–0·41	None	S	0·602	0·604
^*^04	^9^^,^^10^^,^^12^^,^^13^^,^^15^^,^^27^^,^^28^^,^^30^^–^^34^	366/1150	697/5397	66·31	< 0·00001	83	Random	< 0·00001	3·61	2·28–5·71	None	S	0·537	0·645
^*^07	^9^^,^^10^^,^^12^^,^^27^^,^^28^^,^^31^^,^^33^^–^^35^	55/962	289/2436	19·34	0·01	59	Random	0·004	0·45	0·26–0·78	None	S	0·754	0·864
^*^08	^10^^,^^12^^,^^15^^,^^28^^,^^31^^,^^32^^,^^34^	43/630	49/2724	12·94	0·04	54	Random	0·03	2·25	1·07–4·70	Except^15^^,^^31^^,^^32^	S	1·000	0·873
^*^09	^10^^,^^28^^,^^33^^,^^34^	21/378	102/1200	2·70	0·44	0	Fixed	0·07	0·59	0·34–1·04	None	S	0·734	0·670
^*^10	^10^^,^^12^^,^^15^^,^^28^^,^^33^^,^^34^	6/538	44/1482	2·49	0·78	0	Fixed	0·09	0·48	0·21–1·12	None	S	0·296	0·512
^*^11	^10^^,^^12^^,^^15^^,^^28^^,^^31^^,^^33^^,^^34^	75/604	347/2000	2·51	0·87	0	Fixed	< 0·00001	0·47	0·34–0·63	None	S	0·764	0·814
^*^12	^10^^,^^12^^,^^15^^,^^28^^,^^33^^,^^34^	19/538	71/1952	14·25	0·01	65	Random	0·83	0·88	0·27–2·83	None	S	1·000	0·668
^*^13	^10^^,^^12^^,^^15^^,^^28^^,^^31^^,^^33^^,^^34^	35/566	129/1504	2·51	0·87	0	Fixed	0·06	0·68	0·45–1·01	Except^12^^,^^34^	S	1·000	0·654
^*^14	^10^^,^^12^^,^^13^^,^^15^^,^^27^^,^^28^^,^^31^^–^^34^	336/1048	199/3280	24·40	0·004	63	Random	< 0·00001	6·47	4·52–9·26	None	A	0·049	0·034
^*^15	^10^^,^^15^^,^^27^^,^^28^^,^^31^^,^^34^	35/736	216/1594	10·43	0·06	52	Random	0·001	0·35	0·18–0·66	None	S	1·000	0·533
^*^16	^10^^,^^15^^,^^28^^,^^34^	9/402	56/1254	1·56	0·67	0	Fixed	0·05	0·47	0·23–0·98	Except^15^^,^^28^^,^^34^	S	1·000	0·773

*P*h, *P*-value for heterogeneity; *P*c, *P*-value for comparability; OR, pooled odds ratio; CI, confidence interval; S, symmetrical; A, asymmetrical.

**Table 4:** Summary of associations between HLA-DRB1 phenotypes and pemphigus vulgaris

HLA-DRB1 type	Studies	Patients (*n* events/*n* total)	Controls (*n* events/*n* total)	Heterogeneity							Model	Association											Sensitivity analysis	Publication bias														
χ^2^	*P*h	*I*^2^%	*P*c	OR	95% CI	Funnel plot	Begg’s test (*P*r)	Egger’s test (*P*)
^*^01	^14^^,^^16^^,^^17^^,^^29^^,^^31^^,^^35^	17/145	91/571	22·73	0·0004	78	Random	0·70	0·75	0·17–3·34	Except^17^	S	0·452	0·174
^*^03	^16^^,^^17^^,^^29^^,^^31^^,^^32^^,^^35^	8/145	333/1332	6·80	0·24	27	Fixed	0·0001	0·25	0·12–0·51	None	S	0·734	0·619
^*^04	^8^^,^^14^^,^^16^^,^^17^^,^^29^^,^^31^^,^^32^^,^^35^	116/213	360/1902	32·42	< 0·00001	78	Random	0·0002	4·14	1·98–8·65	None	S	0·711	0·894
^*^07	^16^^,^^17^^,^^29^^,^^31^^,^^32^^,^^35^	9/129	255/1036	6·06	0·30	17	Fixed	0·0002	0·27	0·14–0·54	None	S	1·000	0·837
^*^08	^8^^,^^14^^,^^16^^,^^17^^,^^29^^,^^31^^,^^32^^,^^35^	33/181	83/1096	5·18	0·52	0	Fixed	0·0003	2·46	1·51–4·01	None	S	0·707	0·896
^*^09	^8^^,^^14^^,^^16^^,^^17^^,^^35^	4/134	196/983	1·18	0·88	0	Fixed	0·0003	0·19	0·08–0·46	None	S	1·000	–
^*^10	^16^^,^^17^^,^^35^	4/66	16/413	3·25	0·20	39	Fixed	0·25	1·85	0·65–5·23	None	S	–	–
^*^11	^16^^,^^17^^,^^29^^,^^31^^,^^35^	27/113	145/526	3·42	0·49	0	Fixed	0·13	0·68	0·41–1·12	Except^16^	S	0·734	0·595
^*^12	^14^^,^^16^^,^^17^^,^^35^	2/98	24/458	1·19	0·76	0	Fixed	0·39	0·59	0·17–1·97	None	A	–	–
^*^13	^14^^,^^16^^,^^17^^,^^29^^,^^31^^,^^35^	13/145	125/571	6·85	0·23	27	Fixed	0·005	0·42	0·23–0·77	Except^16^	S	0·308	0·123
^*^14	^8^^,^^14^^,^^16^^,^^17^^,^^29^^,^^31^^,^^32^^,^^35^	118/213	179/1902	24·86	0·0008	72	Random	< 0·00001	9·68	4·47–20·98	None	A	0·368	0·548
^*^15	^14^^,^^17^^,^^29^^,^^31^^,^^35^	9/119	73/371	6·12	0·19	35	Fixed	0·002	0·32	0·16–0·65	Except^14^	S	0·221	0·014
^*^16	^17^^,^^29^^,^^35^	3/73	35/315	4·20	0·12	52	Random	0·55	0·55	0·08–3·96	None	S	–	–

*P*h, *P*-value for heterogeneity; *P*c, *P*-value for comparability; OR, pooled odds ratio; CI, confidence interval; S, symmetrical; A, asymmetrical.

In both allele and phenotype analyses, the overall gene frequency of DRB1*04 was significantly higher in patients with PV (31·83% and 54·46%, respectively) than in controls (12·91% and 18·93%, respectively) (*P* ≤0·05). A random-effects model was used based on the detected heterogeneity in the test results (*P*h < 0·00001, *I*^2^ = 83% and *P*h < 0·00001, *I*^2^ = 78%, respectively). Furthermore, stratified meta-analyses (Table 5) and meta-regression analysis (Table 6) were conducted to determine the possible sources of heterogeneity. The ethnicity-stratified test showed reduced heterogeneity for Asians (*I*^2^ = 0%). In the sensitivity analysis, the ORs and 95% CI directions remained stable, suggesting that the results were consistent to some extent.

**Table 5:** Meta-analysis stratified by study quality and ethnicity

HLA–DRB1 type	Ethnicity						Quality			
	Asian		Caucasian		Other		High		Low	
	*I*^2^%	95% CI	*I*^2^%	95% CI	*I*^2^%	95% CI	*I*^2^%	95% CI	*I*^2^%	95% CI
^*^04 genotype	**0**	**1·33–3·86**	87	1·65–7·71	84	1·62–7·05	88	1·78–6·96	76	1·97–7·18
^*^04 phenotype	89	0·65–3·79	46	2·39–4·40	–	1·49–3·32	82	1·84–4·09	62	0·78–2·73
^*^07 genotype	75	0·16–4·22	**16**	**0·19–0·57**	**13**	**0·29–0·92**	68	0·21–0·50	**0**	**0·37–0·93**
^*^08 genotype	–	0·36–2·43	**0**	**2·38–9·68**	0	0·60–2·74	60	0·97–4·67	**–**	0·30–126·18
^*^14 genotype	53	3·00–15·13	56	4·71–13·10	**7**	**3·37–6·81**	66	5·91–9·78	45	3·54–7·88
^*^14 phenotype	86	0·79–124·32	46	4·68–19·39	–	1·68–14·02	76	3·25–23·68	77	2·08–59·37
^*^15 genotype	65	0·05–1·92	58	0·16–1·11	**0**	**0·10–0·43**	53	0·19–0·99	**0**	**0·18–0·66**

CI, confidence interval. Significant results are shown in bold. –, only one study involved.

**Table 6:** Meta-regression analysis on study characteristics

HLA-DRB1 type	Publication year		Ethnicity		Quality	
	Adjusted *R*^2^%	*P* (> |*t*|)	Adjusted *R*^2^%	*P* (> |*t*|)	Adjusted *R*^2^%	*P* (> |*t*|)
^*^04 genotype	−12·67	0·905	2·31	0·286	−12·28	0·908
^*^04 phenotype	−16·70	0·640	29·65	0·141	19·03	0·213
^*^07 genotype	−12·15	0·418	−8·41	0·476	−7·53	0·356
^*^08 genotype	80·10	0·078	−54·90	0·902	–	–
^*^14 genotype	37·32	0·117	2·00	0·424	−1·06	0·274
^*^14 phenotype	55·02	0·094	**100·00**	**0·032**	−42·21	0·918
^*^15 genotype	100·00	0·103	−40·73	0·858	31·88	0·276

Significant results are shown in bold.

DRB1*08 frequencies were significantly higher in patients with PV than in controls in both the allele (*P*c = 0·03, OR 2·25, 95% CI 1·07–4·70) and phenotype (*P*c = 0·0003, OR 2·46, 95% CI 1·51–4·01) analyses, but heterogeneity (*I*^2^ = 54%) was observed in the allele group. Ethnicity-stratified meta-analyses implied that DRB1*08 increased significantly only for caucasians. In the sensitivity analysis, three studies^15^^,^^31^^,^^32^ were determined to impact the statistically significant effect of ORs and 95% CIs. Notably, these three studies all depended on caucasians and were consistent with ethnicity-stratified results in Table 5.

In both allele and phenotype analyses, DRB1*14 showed a remarkably high risk for PV occurrence (*P*c < 0·00001, OR 6·47, 95% CI 4·52–9·26 and *P*c < 0·00001, OR 9·68, 95% CI 4·47–20·98, respectively). However, significant heterogeneity (*I*^2^ = 63% and *I*^2^ = 72%, respectively) was found to exist among the studies. Meta-regression analysis (Table 6) revealed that different ethnicities were a significant source of heterogeneity (adjusted *R*^2^ of DRB1*14 phenotype = 100%, *P* =0·032). The comparability of results did not change after a sensitivity analysis was conducted by sequentially omitting each study.

DRB1*03 showed a strong protective effect against PV in both the allele (*P*c < 0·00001, OR 0·28, 95% CI 0·19–0·41) and phenotype (*P*c = 0·0001, OR 0·25, 95% CI 0·12–0·51) analyses. No significant heterogeneity was detected in either analysis (*I*^2^ = 0% and *I*^2^ = 27%, respectively).

Both DRB1*07 and DRB1*15 were significantly negatively correlated to the incidence of PV. Nevertheless, high-level heterogeneity (DRB1*07, *I*^2^ = 59%; DRB1*15, *I*^2^ = 52%) occurred in the allele analyses, despite the random-effects model having been used. Stratified meta-analyses (Table 5) and meta-regression analyses (Table 6) were carried out for both alleles and phenotypes. For DRB1*07, the ethnicity-stratified analysis showed a reduction of heterogeneity in caucasians (*I*^2^ = 16%) and other mixed ethnicities (*I*^2^ = 13%). Moreover, there was not significant heterogeneity (*I*^2^ = 0%) among the studies assessed as low quality. For DRB1*15, results were consistent among studies of other ethnicities (*I*^2^ = 0%) and those of low quality (*I*^2^ = 0%). A sensitivity analysis revealed that the DRB1*15 phenotype data in Niizeki *et al.*^14^ influenced comparability.

DRB1*01, DRB1*09, DRB1*13 and DRB1*16 were not statistically significant in either the allele or phenotype analyses, and produced several unstable outcomes in sensitivity analysis. None of the results varied obviously in stratification or meta-regression analysis of studies by geography or study quality.

### Publication bias

All the funnel plots were inspected for geometrical symmetry and most were found to be roughly symmetrical. Only the plots of DRB1*12 phenotype, and DRB1*14 allele and phenotype were asymmetrical. The funnel plots confirmed the absence of publication bias (Tables 3 and 4), which was in accordance with outcomes of Begg’s and Egger’s tests (*P* >0·05). DRB1*14 was the only notable exception, with *P* =0·049 for Begg’s test and *P* =0·034 for Egger’s test. The publication bias of DRB1*10/12/16 phenotypes was incalculable, with only one article left due to automatic rejection of zero events in the arithmetic.

## Discussion

Extensive research has been carried out to determine the function of HLA in the pathogenesis of PV; however, the findings to date have been inconsistent and controversial. Therefore, we performed a meta-analysis to investigate more comprehensively the potential association between HLA-DRB1 and PV, the first of its kind in the literature, to our knowledge.

Our study revealed that DRB1*04, DRB1*08 and DRB1*14 were susceptibility factors for PV, whereas DRB1*03, DRB1*07 and DRB1*15 may be negatively associated with PV across racial lines. When the patients and controls were stratified into subgroups by ethnicity, DRB1*07 frequencies were found to be significantly lower in caucasian and other mixed ethnicities and DRB1*08 was obviously increased in caucasians. In addition, DRB1*04, DRB1*14 and DRB1*15 were strongly correlated with PV incidence, despite remarkable heterogeneity. Meta-regression analysis by ethnicity and study quality could not explain the between-study variance assessed by Knapp–Hartung modification (*P* >0·05) in STATA/SE 11.2 software. According to the majority of earlier studies, pemphigus is most often associated with DR4 and DR14, regardless of ethnicity.^12^^,^^16^^,^^32^^,^^36^^,^^36^^–^^39^ For example, molecular-based genotyping^18^ revealed an association with DRB1*0402 in Ashkenazi Jews,^9^ non-Jewish Iranians,^34^ French,^13^ Italians,^15^ Spaniards,^16^ Sardinians^32^ and Tunisians.^37^ DRB1*0403, *0404 and *0406, however, were associated with PV in Japanese individuals.^8^ Likewise, DRB1*1401 was correlated with PV in French,^13^ Italian,^15^ Japanese^8^^,^^14^ and Spanish^16^ populations, while DRB1*1404 was correlated with PV in eastern Indian and Pakistani subjects.^12^ In addition, different genotypes have been shown to be associated with PV in different studies of the same population.^8^^,^^14^^,^^38^ Therefore, subtype–gene analysis among populations from disparate nations^40^ may help to uncover further the sources of heterogeneity. DRB1*04:02,^9^ for instance, is clearly associated with PV in Ashkenazi Jews, and shows the highest frequencies. DRB1*0802 and *1408^14^ are associated with Japanese patients with PV, and the highest frequencies occur in eastern Asia. DRB1*04:06^10^^,^^11^^,^^38^ and *14:05^11^^,^^14^^,^^38^ are frequent both in Chinese and in Japanese.

Notably, strong linkage disequilibrium is reported to exist across DR–DQ loci with PV, such as DQB1*0503–DRB1*1401,^41^ DRB1*04–DQB1*03^8^^,^^18^ and DRB1*04–DQA1*0301.^8^^,^^42^ However, it is still unclear whether over/underrepresented haplotypes represent a true association. Lee *et al.*^42^ found that both haplotypes {DRB1*0402; DQB1*0302} and {DRB1*0402; not DQB1*0302} are significantly elevated in patients with PV. However, when DRB1*0402 is excluded from the haplotype {not DRB1*0402; DQB1*0302}, the frequency of this haplotype is not significantly different between patients and controls. Considering the variability and quantity of relevant haplotypes, the role of linkage disequilibrium was not addressed in our study.

Although the correlation of PV with HLA class II genes has been demonstrated by various studies, the mechanisms underlying the effect have yet to be elucidated. It is known that the interaction of anti-Dsg3 antibodies with their target antigens is responsible for loss of cell adhesion (acantholysis). The loss of tolerance to target autoantigens is considered to involve HLA class II molecules, the initiating autoantigenic peptide(s), and T-cell receptors (TCRs).^43^ In PV, the interaction between the TCR and the major histocompatibility complex (MHC)–peptide complex may be a key step towards triggering T-cell ability to respond to Dsg3, especially for the CD4+ subtype. Many reports have shown that HLA-DR molecules can bind multiple self-peptides with high efficiency and that DR molecules may act as restrictive elements for peptide presentation.^13^ The HLA-DR β1 chain^44^ gene is localized in the MHC class II region that encodes immunomodulatory factors involved in recognition of extracellular proteins and autoantigens. Nonconservative substitutions of key amino acid residues in the hypervariable region (α1 and β1) coded by the HLA-DRB1 gene mediate variations in the HLA class II molecule space structure. Put more precisely, changes in the primary amino acid sequence can affect the pocket surface electrostatic potential and modify protein function^6^ through steric hindrance. Such differentially targeted binding potency could result in a variable antigen-specific immune response to various Dsg family members, such as Dsg3. In fact, both abnormal antigen presentation and T-cell dysfunction are known to be involved in PV pathogenesis.^45^ For example, autoreactive T cells of patients with PV recognized distinct Dsg3 peptides with conserved anchor motifs required for binding to some HLA-DRB1 alleles.^7^ However, as PV does not result from the aberrant presentation of a single product of the HLA gene, more complex immune mechanisms, such as cytokines secreted by Dsg3-reactive Th cells involved in B-cell differentiation and Dsg-specific autoantibody production,^5^ may also promote PV onset and progression.

Meta-analysis is an established and effective strategy to increase the sample size by pooling data from individual studies to enhance the statistical power of the analysis.^46^ However, in our meta-analysis several limitations exist that may impact the interpretation of our results. First, many of the studies involved in our meta-analysis did not control for matching variables, such as age, sex or ethnicity. Each of these could have confounded the studies’ estimates. Second, nonsignificant results are less likely to be accepted for publication and inclusion among the searchable literature. Any type of missing data can cause false-positive results. Although we performed Begg’s and Egger’s regression tests and found no significant publication bias, we cannot completely rule out the possibility of disequilibrium.^47^^,^^48^ Third, as with other complex autoimmune diseases, PV is likely to have a multifactorial cause, possibly including the combination of different genes, environmental effects and the real prevalence of each variant in the population. We did not analyse the complex interplay between various genes due to limited data, and our conclusions were drawn without considering environmental or inherent factors. Fourth, HLA genotyping techniques have different sensitivities. For example, results from polymerase chain reaction with probe hybridization do not always coincide with sequencing results. Yet, we did not include the particular methodologies used in our meta-analysis. Fifth, some relevant published or unpublished data may be overlooked or beyond the scope of our discovery strategy. Finally, pemphigus is a relatively rare and severe disease. As such, it is difficult for clinicians to conduct large-scale randomized controlled trials. The case–control studies used in our meta-analysis were based on small samples and were of lower scientific evidence level, which might have decreased our ability to detect a difference in the distribution of HLA-DRB1.

Nevertheless, research to determine the primary role of some regions of the HLA locus in the pathogenesis of PV may have value in subsequent prospective studies to uncover the underlying mechanisms. Ethnic-specific PV alleles may influence the susceptibility or resistance to PV by attributing to a direct involvement of the HLA molecules as an antigen presenter or a neighbouring linked gene.^49^ In addition, our identification of some significant risk and protective genotypes/haplotypes among various ethnicities in heterogeneous geographical regions illustrated that such genetic variants are likely to be shared with other populations of the world. However, to confirm our observations it is necessary to conduct large-scale clinical studies with standardized blinding methods, homogeneous PV patient samples and well-matched controls. In conclusion, our results provide some insights into the correlation of HLA-DRB1 with PV but should be considered cautiously with respect to the limitations of our study and the underlying data.

## What’s already known about this topic?

- Various alleles of the HLA-DRB1 gene have been associated with pemphigus vulgaris (PV), but studies of these genes have been largely carried out in small populations and the results from different studies have not been consistent.

## What does this study add?

- To the best of our knowledge, this is the first meta-analysis on the association between HLA-DRB1 and PV.
- DRB1*04, DRB1*08 and DRB1*14 may act as susceptibility factors for PV, while DRB1*03, DRB1*07 and DRB1*15 were found to be significantly negatively associated with PV.
- The roles of DRB1*07, DRB1*08 and DRB1*14 phenotypes are partially dependent on ethnicity.
